# tRNA m^1^A modification is essential for gut homeostasis and function of group 3 innate lymphoid cells

**DOI:** 10.1038/s41421-025-00850-9

**Published:** 2026-01-03

**Authors:** Jingyu Li, Zirun Tang, Yunzhu Chen, Xuemin Cai, Longyan Wu, Gaoyang Wang, Chen Kan, Bin Li, Bing Su, Huabin Li, Coco Chu, Hua-Bing Li

**Affiliations:** 1 Institute for Immunology and Pathogenesis, Chongqing Medical University, Chongqing, China; 2 Medical Center on Aging, Center for Immune-Related Diseases at Shanghai Institute of Immunology, Ruijin Hospital, Shanghai Jiao Tong University School of Medicine, Shanghai, China; 3 Shanghai Jiao Tong University School of Medicine-Yale Institute for Immune Metabolism, Shanghai Jiao Tong University School of Medicine, Shanghai, China; 4 Institute for Immunology, Beijing Key Laboratory of Immunological Research of Allergy, School of Basic Medical Sciences, Tsinghua-Peking Center for Life Sciences, School of Medicine, Tsinghua University, Beijing, China; 5 School of Biological Science, The University of Manchester, Manchester, UK; 6 ENT institute and Department of Otorhinolaryngology, Eye & ENT Hospital, Fudan University, Shanghai, China; 7 SXMU-Tsinghua Collaborative Innovation Center for Frontier Medicine, Shanxi Medical University, Taiyuan, Shanxi China; 8 Digestive Diseases Center, Beijing Tsinghua Changgung Hospital, Beijing, China; 9 Chongqing International Institute for Immunology, Chongqing, China

**Keywords:** Innate immunity, RNA modification

## Abstract

Group 3 innate lymphoid cells (ILC3s) play crucial roles in maintaining intestinal homeostasis and defending against bacterial infections. However, the epigenetic mechanisms that regulate ILC3 responses are not well understood. In this study, we show that *Trmt61a*, the methyltransferase responsible for the m^1^A58 tRNA modification, is predominantly expressed in ILC3s. We found that specific depletion of TRMT61A in ILC3s leads to dysregulated cell cycle and a reduction in cell numbers. Notably, mice with an ILC3-specific TRMT61A deficiency exhibit dysbiosis, but antibiotic treatment can restore colonic ILC3 levels. Furthermore, these mice exhibit increased susceptibility to experimental intestinal inflammation and enteric bacterial infection. Our findings uncover a previously unrecognized role for TRMT61A mediated m^1^A modification in the regulation of intestinal ILC3s, essential for protecting intestinal tissue during inflammation and enhancing innate immunity against enteric pathogens.

## Introduction

Innate lymphoid cells (ILCs) constitute a family of innate immune cells lacking antigen-specific receptors characteristic of T cells and B cells^1–4^. These cells are categorized into three groups based on their master transcription factors and cytokine profiles. Group 1 ILCs (ILC1s) express T-bet and produce IFN-γ, group 2 ILCs (ILC2s) express GATA3 and secrete type 2 cytokines such as interleukin (IL)-5 and IL-13, while group 3 ILCs (ILC3s) express RORγt and generate IL-17 and IL-22^1–4^. ILC3s are primarily found in mucosal tissues like the intestinal lamina propria. Unlike T cells, which require priming and migration to inflamed tissues^5^, ILC3s are tissue resident^6^ and are quickly activated by pro-inflammatory cytokines such as IL-23, IL-1β and TNF-like factor 1A (TL1A), secreted by mononuclear phagocytes^6–10^

ILC3s are critical in intestinal immunity, particularly during injury and inflammation^8,9,11^. They produce IL-22, which activates the STAT3 signaling in epithelial cells, enhancing their regeneration and proliferation^11^. In the initial stages of bacterial infections, ILC3s are the primary source of IL-22, which induces the expression of antimicrobial peptides such as RegIIIβ and RegIIIγ, providing protection against the infection^12–14^. This process is intricately modulated at both transcriptional levels and epigenetic levels, including DNA and histone modifications. Research has highlighted the role of DNA hydroxymethylase tet methylcytosine dioxygenase 2 (TET2) in controlling ILC3 functions through the distribution of methyl-CpG and 5- hydroxymethylcytosine (5hmC)^15^. Additionally, the histone H3K36 methyltransferase SET domain containing 2 (SETD2) affects genome accessibility, impacting the maintenance and function of ILC3 subsets^16^. Yet, the impact of RNA modifications on ILC3s is less understood.

Among the more than 100 identified post-transcriptional RNA modifications, N1- methyladenosine (m^1^A) methylation is one of the most conserved and abundant^17,18^. m^1^A modification, particularly m^1^A58 in tRNA, is crucial for maintaining tRNA stability and enhancing protein translation initiation and elongation^19^. The m^1^A methyltransferase complex, consisting of the catalytic subunit TRMT61A and the non- catalytic subunit TRMT6, catalyzes this modification, which could be reversed by the demethylase ALKBH1^20^. While m^1^A modifications are linked to the progression of various cancers^21–23^, their roles in the immune system, particularly within ILC3s, remain poorly defined.

In this study, we demonstrated that *Trmt61a* is primarily expressed by intestinal ILC3s. Genetic deletion of ILC3-intrinsic TRMT61A led to reduced numbers of total and IL-22-producing ILC3s, alongside dysregulated cell proliferation and apoptosis. Furthermore, dysbiosis in *Trmt61a*^*ΔRorc*^ mice, which could be largely corrected with antibiotics, exacerbated these effects. These findings underscore a novel role for TRMT61A mediated tRNA m^1^A modification in regulating ILC3s, essential for their protective immunity in intestinal health and defense against bacterial infections.

## Results

### *Trmt61a* expression and its role in ILC3 homeostasis in the intestine

To investigate the role of tRNA m^1^A58 modification in the immune system, we first reanalyzed existing single-cell RNA sequencing (scRNA-seq) data of CD45^+^ immune cells isolated from the small intestine^24^. We found that *Trmt61a* was primarily expressed in ILC3s, dendritic cells (DCs), and plasmacytoid DCs (pDCs), with the highest expression levels observed in ILC3s (Supplementary Fig. S1a–c).

**Fig. 1:**
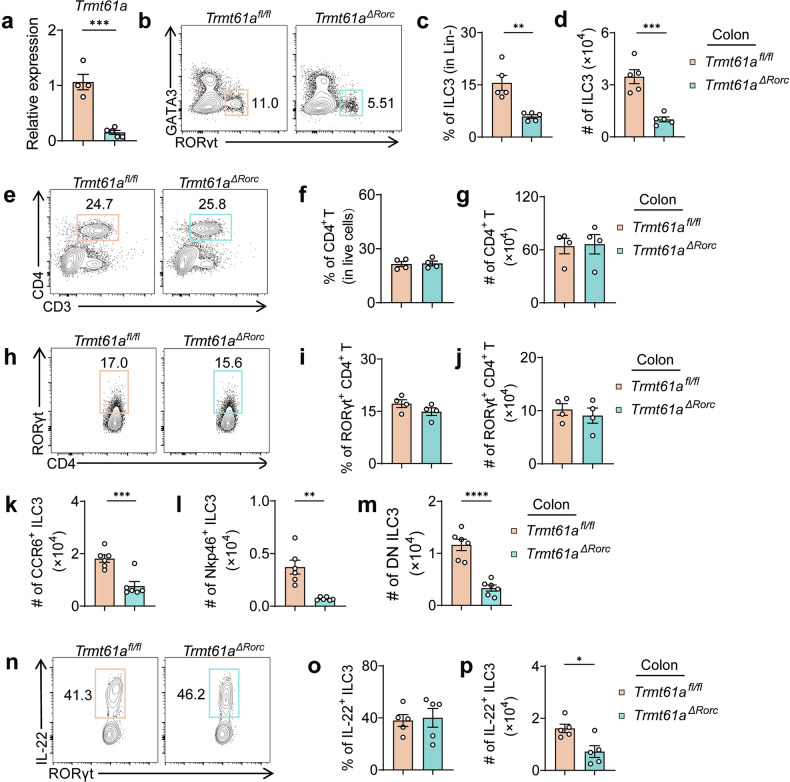
*Trmt61a* Expression and its impact on ILC3s and intestinal microbial homeostasis. **a** Real-time RT-PCR analysis of *Trmt61a* mRNA levels in ILC3s isolated from the colonic lamina propria of *Trmt61a*^*fl/fl*^ (*n* = 4) and *Trmt61a*^*ΔRorc*^ mice (*n* = 5). **b**–**d** Flow cytometry analysis of ILC3s in colonic lamina propria lymphocytes (LPLs): representative plots (**b**); population frequency (**c**); cell counts (**d**). *n* = 5 mice per group. **e**–**j** Analysis of CD4^+^ and RORγt^+^ CD4^+^ T cells in colonic LPLs: **e**, **h** Representative flow cytometry plots. **f**, **i** Population frequencies. **g**, **j** Cell counts. *n* = 4 mice per group. **k**–**m** Counts of ILC3 subsets in colonic LPLs: **k** CCR6^+^ ILC3s. **l** Nkp46^+^ ILC3s. **m** DN (double-negative) ILC3s. *n* = 6 mice per group. **n**–**p** IL-22^+^ ILC3s in colonic LPLs: **n** Representative flow cytometry plots. **o** Population frequency. **p** Cell counts. *n* = 5 mice per group. Data are pooled from two independent experiments, presented as means ± SEM, *p < 0.05, **p < 0.01, ***p < 0.001, and ****p < 0.0001.

To study the function of *Trmt61a* and tRNA m^1^A modification in ILC3 biology, we crossed *Trmt61a*^*fl/fl*^ mice with *Rorc*^*cre*^ mice to specifically delete *Trmt61a* expression in ILC3s. The resulting *Rorc*^*cre*^*Trmt61a*^*fl/fl*^ mice, referred to as *Trmt61a*^*ΔRorc*^ mice, exhibited significantly reduced expression of *Trmt61a* in ILC3s from both small intestine and colon (Fig. 1a; Supplementary Fig. S2a). Notably, even under steady-state conditions, *Trmt61a*^*ΔRorc*^ mice already displayed significantly decreased percentages and numbers of ILC3s in the intestine compared to control *Trmt61a*^*fl/fl*^ mice (Fig. 1b–d; Supplementary Figs. S1d, S2b–d). There were no observed differences in the percentages and numbers of total CD4^+^ T cells and RORγt^+^CD4^+^ T cells (Fig. 1e–j; Supplementary Fig. S2e–j). Furthermore, *Trmt61a*^*ΔRorc*^ mice exhibited significantly decreased numbers of all three subsets of ILC3s — NKp46^+^ ILC3s, CCR6^+^ ILC3s, and NKp46^–^CCR6^–^ double-negative (DN) ILC3s — compared to control *Trmt61a*^*fl/fl*^ mice (Fig. 1k–m; Supplementary Fig. S2k–m). Additionally, although the percentages remained unchanged, the numbers of IL-22-producing ILC3s were significantly lower in the intestine of *Trmt61a*^*ΔRorc*^ mice compared to controls (Fig. 1n–p; Supplementary Fig. S2n–p). These findings underscore the critical role of TRMT61A in maintaining the homeostasis of all ILC3 subsets in the intestine.

Additionally, to determine whether the decreased numbers of ILC3s in *Trmt61a*^*ΔRorc*^ mice result from developmental defects, we generated bone marrow chimeras by reconstituting lethally irradiated CD45.1^+^ wild-type recipients with a 1:1 mixture of CD45.2^+^ *Trmt61a*^*ΔRorc*^ (or *Trmt61a*^*fl/fl*^ control) and CD45.1^+^ wild-type bone marrow (Supplementary Fig. S3a). Eight weeks post-transplantation, the CD45.2/CD45.1 ratios within intestinal ILC3s were comparable between the two groups (Supplementary Fig. S3b, c). These results suggest that the reduction of ILC3s in *Trmt61a*^*ΔRorc*^ mice is not due to developmental defects.

### Impact of TRMT61A on proliferation and apoptosis in intestinal ILC3s

**Fig. 2:**
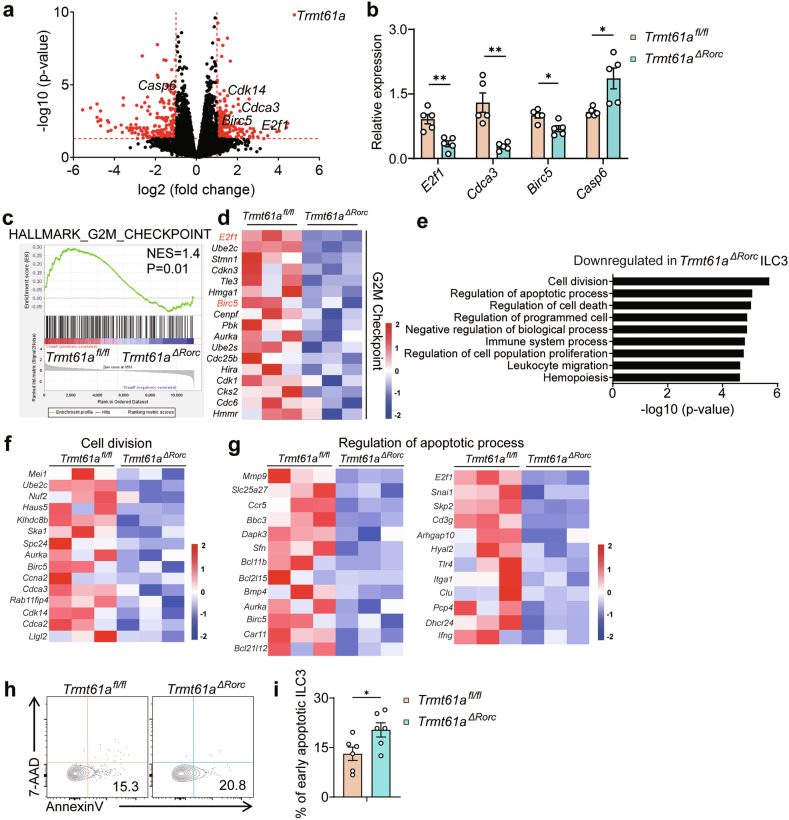
TRMT61A regulation of proliferation and apoptosis of ILC3s. ILC3s (Lin^–^CD45.2^int^CD90.2^hi^) were isolated from the colonic LPLs of *Trmt61a*^*fl/fl*^ and *Trmt61a*^*ΔRorc*^ mice. The mRNA from triplicate samples of purified ILC3s was extracted and subjected to genome-wide transcriptome analysis (RNA-seq). **a** Differential gene expression between *Trmt61a*^*fl/fl*^ and *Trmt61a*^*ΔRorc*^ ILC3s is displayed. Red points indicate genes with significant differences in expression. **b** Real-time RT-PCR analysis of specific gene expression in *Trmt61a*^*fl/fl*^ and *Trmt61a*^*ΔRorc*^ ILC3s. *n* = 5 mice per group. **c** Gene-set enrichment analysis (GSEA) illustrated that genes associated with the G2- M checkpoint pathway were expressed at higher levels in *Trmt61a*^*fl/fl*^ ILC3s compared to *Trmt61a*^*ΔRorc*^ ILC3s. **d** Heat map displaying the expression of transcripts associated with the G2-M checkpoint pathway in *Trmt61a*^*ΔRorc*^ ILC3s vs *Trmt61a*^*fl/fl*^ ILC3s. **e** Gene ontology (GO) enrichment analysis was performed on genes downregulated more than 2-fold in *Trmt61a*^*ΔRorc*^ ILC3s, highlighting representative enriched biological processes. **f**, **g** Heat maps showing lists of downregulated genes related to cell division (**f**) and the regulation of the apoptotic process (**g**) in *Trmt61a*^*ΔRorc*^ ILC3s. **h**, **i** Early apoptotic ILC3s in the colon of *Trmt61a*^*fl/fl*^ and *Trmt61a*^*ΔRorc*^ mice were assessed by Annexin V/7-AAD staining. Representative flow cytometry plots showing AnnexinV⁺/7-AAD⁻ ILC3s (**h**). Quantification of early apoptotic ILC3s (**i**). *n* = 6 mice per group. Data are pooled from two independent experiments, presented as means ± SEM. *p < 0.05, **p < 0.01.

To explore the underlying molecular mechanism by which TRMT61A regulates ILC3 homeostasis, we isolated ILC3s from the intestines of *Trmt61a*^*ΔRorc*^ and *Trmt61a*^*fl/fl*^ mice and performed transcriptome sequencing. The RNA-seq analysis revealed 151 genes significantly downregulated and 195 genes significantly upregulated in *Trmt61a-*deficient ILC3s compared to control (Fig. 2a). Notably, genes associated with the cell cycle and proliferation, such as *Ccna2*^25^, *Cdca3*^26^, and *E2f1*^27^ were significantly downregulated in *Trmt61a*-deficient ILC3s (Fig. 2a). Additionally, the expression of the pro-apoptotic gene *Casp6*^28^ was significantly increased, while the anti-apoptotic gene *Birc5*^29^ was markedly reduced in these cells (Fig. 2a). Quantitative PCR further validated these findings, confirming reduced expressions of *E2f1, Cdca3*, and *Birc5*, and increased expression of *Casp6* in *Trmt61a-*deficient ILC3s compared to controls (Fig. 2b).

Gene set enrichment analysis (GSEA) demonstrated that the G2/M checkpoint signature was enriched in control ILC3s but absent in TRMT61A-deficient cells (Fig. 2c, d). Furthermore, gene ontology (GO) analysis of downregulated genes in TRMT61A-deficient ILC3s identified pathways related to cell division and the regulation of the apoptotic process (Fig. 2e–g). Consistent with these transcriptional changes, Annexin V/7-AAD staining of intestinal ILC3s showed a significant increase in early apoptotic cells in *Trmt61a*^*ΔRorc*^ mice compared with controls (Fig. 2h, i). These findings collectively indicate that TRMT61A plays a critical role in modulating genes transcription related to proliferation and apoptosis, thereby maintaining the homeostasis of ILC3s in the intestine.

### The microbiota modulates TRMT61A-mediated ILC3 homeostasis

**Fig. 3:**
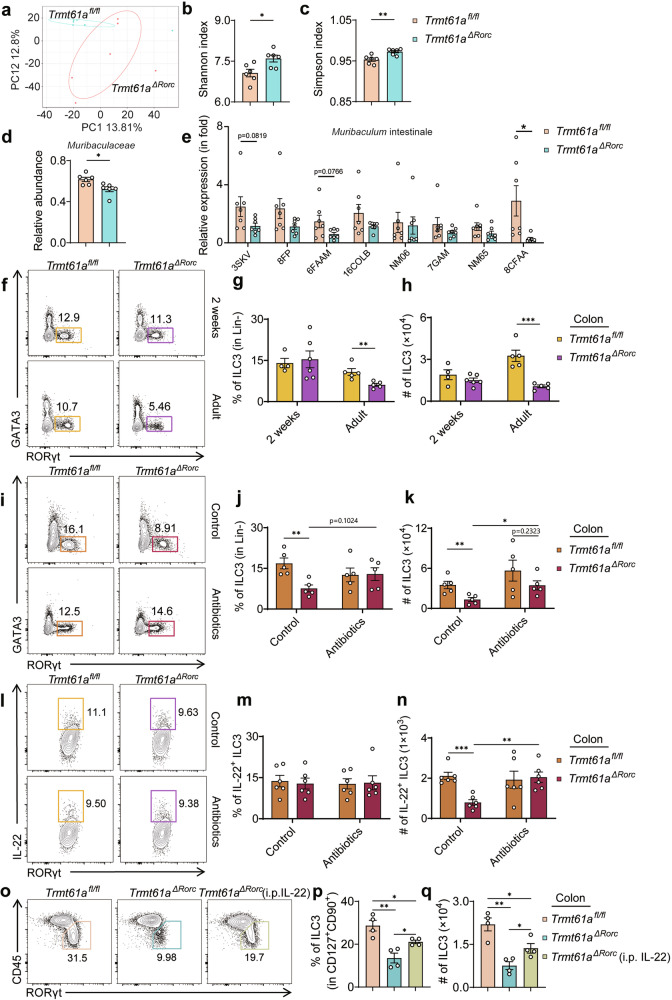
Involvement of microbiota in the regulation of TRMT61A in ILC3s. **a**–**d** Fecal microbiota analysis of *Trmt61a*^*fl/fl*^ and *Trmt61a*^*ΔRorc*^ mice using 16S rRNA gene sequencing: principal coordinates analysis (PCA) depicting the overall microbial composition (**a**), Shannon-Wiener diversity index (Shannon index) of fecal samples (**b**), Simpson’s Diversity Index (Simpson index) of fecal samples (**c**), relative abundance of *Muribaculaceae* in fecal samples (**d**). *n* = 6 mice. **e** Fecal pellets were collected, and total bacterial DNA was extracted. Quantitative PCR was then performed for specific bacteria, with normalization against total bacterial DNA. *n* = 7 mice per group. **f**–**h** Analysis of ILC3 populations in colonic LPLs of 2- week-old and adult *Trmt61a*^*fl/fl*^ and *Trmt61a*^*ΔRorc*^ mice: representative flow cytometry plots (**f**), population frequencies of ILC3s (**g**), counts of ILC3s (**h**). *n* = 4 to 6 mice per group. **i**–**k** Effects of antibiotic treatment on ILC3 populations in colonic LPLs of *Trmt61a*^*fl/fl*^ and *Trmt61a*^*ΔRorc*^ mice, 2 months post-treatment: representative flow cytometry plots (**i**), population frequencies of ILC3s (**j**), counts of ILC3s (**k**). *n* = 5 mice per group. **l**–**n** Analysis of IL-22^+^ ILC3s in the colon of *Trmt61a*^*fl/fl*^ and *Trmt61a*^*ΔRorc*^ mice with or without antibiotic treatment: representative flow cytometry plots (**l**), population frequency of IL-22^+^ ILC3s (**m**), cell counts of IL-22^+^ ILC3s (**n**). *n* = 6 mice per group. **o**–**q** Analysis of ILC3 in the colon of *Trmt61a*^*fl/fl*^ mice, *Trmt61a*^*ΔRorc*^ mice, and *Trmt61a*^*ΔRorc*^ mice with intraperitoneal IL-22 administration: representative flow cytometry plots (**o**), population frequencies of ILC3s (**p**), counts of ILC3s (**q**). *n* = 4 mice per group. Data are presented as means ± SEM. *p < 0.05, **p < 0.01, ***p < 0.001.

The complex interplay between ILC3s and the microbiota is crucial for maintaining intestinal homeostasis^30–32^. Disruptions to this balance, such as through blocking IL-22 or depleting ILC3s, lead to intestinal epithelial dysfunction and dysbiosis^32^. To determine whether dysbiosis occurred in *Trmt61a*^*ΔRorc*^ mice, we conducted 16S rRNA sequencing on fecal samples from both *Trmt61a*^*ΔRorc*^ and *Trmt61a*^*fl/fl*^ mice. The fecal microbiota of *Trmt61a*^*ΔRorc*^ mice showed significant compositional changes compared to controls (Fig. 3a), and displayed greater alpha-diversity, as indicated by the Shannon and Simpson indices^33^ (Fig. 3b, c). This increase in diversity in *Trmt61a*^*ΔRorc*^ mice aligns with findings in IL-22-deficient mice^34^.

At the family level, there was a notable reduction in *Muribaculaceae* in the fecal samples of *Trmt61a*^*ΔRorc*^ mice (Fig. 3d). To validate the 16S rRNA sequencing result and also determine changes of specific *Muribaculaceae* strains, we performed real-time PCR and checked the relative abundance of different *Muribaculaceae* strains in the fecal samples from *Trmt61a*^*fl/fl*^ and *Trmt61a*^*ΔRorc*^ mice. The abundance of *Muribaculum intestinale* 8CFAA was significantly reduced in the fecal samples of *Trmt61a*^*ΔRorc*^ mice, and there was also a trend toward reduced levels of *Muribaculum intestinale* 3SKV and 6FAAM (Fig. 3e). *Muribaculaceae* are key utilizers of monosaccharides in the intestine, essential for producing short-chain fatty acids (SCFAs), which have been shown to significantly influence the proliferation and function of intestinal ILC3s^35–37^. This suggests that the microbiota, via SCFAs, may play a role in modulating TRMT61A activity in ILC3s.

We also examined how weaning impacts the microbiota and ILC3 populations. Weaning is a critical period for microbial diversification due to dietary changes and environmental shifts^38–40^. Upon analyzing ILC3 populations in pre-weaning 2-week-old and adult 8-week-old *Trmt61a*^*ΔRorc*^ mice, in contrast to the deficiency of ILC3s observed in adult *Trmt61a*^*ΔRorc*^ mice, we found that young *Trmt61a*^*ΔRorc*^ mice had similar ILC3 percentages and numbers as their young *Trmt61a*^*fl/fl*^ counterparts (Fig. 3f–h; Supplementary Fig. S4a–c). More interestingly, antibiotic treatment in adult mice normalized the total ILC3 and IL-22^+^ ILC3 counts in *Trmt61a*^*ΔRorc*^ mice to levels seen in adult *Trmt61a*^*fl/fl*^ mice (Fig. 3i–n; Supplementary Fig. S4d–i). underscoring the significant role of microbiota in regulating ILC3 homeostasis mediated by TRMT61A. Moreover, intraperitoneal administration of IL-22 partially restored ILC3 numbers in *Trmt61a*^*ΔRorc*^ mice (Fig. 3o–q; Supplementary Fig. S4j–l), further supporting a role for IL-22 in mediating ILC3 homeostasis.

To investigate the causal relationship between TRMT61A deficiency, microbiota composition, and ILC3 dysfunction, we performed fecal microbiota transfer (FMT) from *Trmt61a*^*ΔRorc*^ or *Trmt61a*^*fl/fl*^ donor mice into antibiotic-treated wild-type recipients, following established protocols^41^. Six weeks after transplantation, we analyzed intestinal ILC3s (Supplementary Fig. S5a). We found that recipients of *Trmt61a*^*ΔRorc*^-derived microbiota (termed FMT-*Trmt61a*^*ΔRorc*^) exhibited a significant reduction in both the percentage and total number of intestinal ILC3s compared with recipients of *Trmt61a*^*fl/fl*^-derived microbiota (termed FMT-*Trmt61a*^*fl/fl*^), along with decreased numbers of IL-22^+^ ILC3s (Supplementary Fig. S5b–m). These findings suggest that microbial dysbiosis in *Trmt61a*^*ΔRorc*^ mice alters the intestinal microenvironment and further exacerbates the reduction of ILC3s.

### TRMT61A is essential for ILC3-mediated protective immunity

**Fig. 4:**
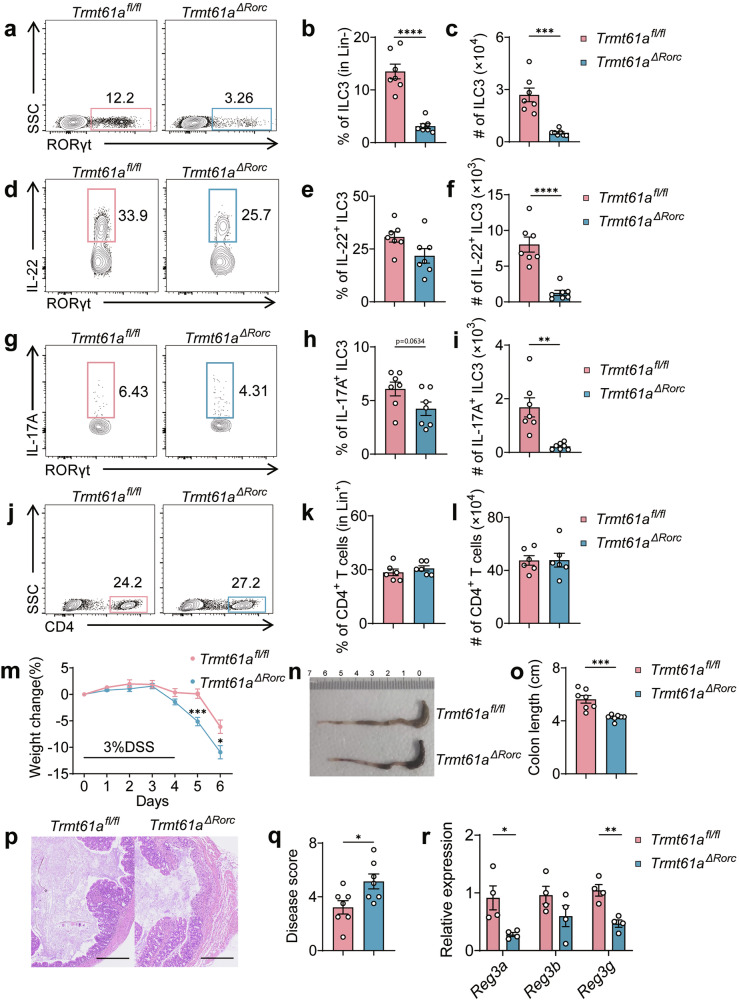
TRMT61A-Mediated protection of ILC3s from acute intestinal damage and inflammation. *Trmt61a*^*fl/fl*^ and *Trmt61a*^*ΔRorc*^ mice were administered 3% dextran sulfate sodium (DSS) for 4 days, followed by regular drinking water for 2 days. Analyses were conducted on day 6 post-initiation of treatment. **a**–**i** Flow cytometry analysis: Representative flow cytometry plots showing total ILC3s (**a**), IL22^+^ ILC3s (**d**) and IL-17A^+^ ILC3s (**g**). Population frequencies of total ILC3s (**b**), IL-22^+^ ILC3s (**e**) and IL-17^+^ ILC3s (**h**). Counts of total ILC3s (**c**), IL-22^+^ ILC3s (**f**) and and IL-17A^+^ ILC3s (**i**) in colonic LPLs of treated mice. *n* = 7 mice per group. **j**–**l** CD4^+^ T Cells analysis in colonic LPLs: representative flow cytometry plots (**j**), population frequency (**k**), cell counts (**l**). *n* = 6 mice per group. **m**–**q** Morphological and histological assessments: body weight changes (**m**), representative photograph of the large intestine (**n**), measurements of colon length (**o**), representative images of colon sections (**p**), Scale bars represent 250 μm. Histology scores assessing tissue damage (**q**). *n* = 7 mice per group. **r** Gene Expression Analysis: real-time RT-PCR was used to analyze the expression of barrier function-related genes in intestinal epithelial cells. *n* = 4 mice per group. Data are pooled from two independent experiments, presented as means ± SEM. *p < 0.05, **p < 0.01, ***p < 0.001, ****p < 0.0001.

ILC3s play critical roles in maintaining intestinal epithelial homeostasis and defending against bacterial infections^42,43^. IL-22, secreted by ILC3s, is crucial for stimulating the production of antimicrobial peptides from intestinal epithelial cells, thereby maintaining barrier integrity^40,43^. To investigate the impact of ILC3-intrinsic TRMT61A on tissue repair during intestinal inflammation, we administered dextran sulfate sodium (DSS) to *Trmt61a*^*ΔRorc*^ and *Trmt61a*^*fl/fl*^ mice. Our findings revealed significantly decreased percentages and numbers of total ILC3s and IL-22^+^ ILC3s in the colons of *Trmt61a*^*ΔRorc*^ mice compared to controls (Fig. 4a–f). The numbers of IL-17A^+^ ILC3s were also significantly decreased in the colons of *Trmt61a*^*ΔRorc*^ mice (Fig. 4g–i). Noteworthily, the counts of CD4^+^ T cells remained similar between the two groups (Fig. 4j–l), indicative of non-involvement of CD4 counterpart. In accordance with the defects of total ILC3s and IL-22^+^ ILC3s, *Trmt61a*^*ΔRorc*^ mice exhibited increased weight loss, greater colonic shortening, and more severe disease pathology scores post-DSS treatment than control mice (Fig. 4m–q). Correspondingly, these mice exhibited reduced expression of IL-22 downstream genes, *Reg3a* and *Reg3g*^12–14^ in epithelial cells from *Trmt61a*^*ΔRorc*^ mice compared to controls (Fig. 4r). Together, these data underscore the essential role of TRMT61A in ILC3-mediated tissue protection during intestine inflammation.

Given that antibiotic treatment partially rescued ILC3 defects in *Trmt61a*^*ΔRorc*^ mice under steady-state conditions (Fig. 3i–n) and in the DSS colitis model (Supplementary Fig. S6a–f), we next assessed whether modulation of the microbiota could mitigate disease severity. *Trmt61a*^*ΔRorc*^ mice were pretreated with a broad-spectrum antibiotic cocktail prior to DSS administration. Consitent with the changes observed in ILC3s, antibiotic treatment also partially ameliorated colitis in *Trmt61a*^*ΔRorc*^ mice, as reflected by reduced weight loss, improved colon length, and lower histological scores (Supplementary Fig. S6g–j). These results indicate that modulation of the intestinal microbiota can partially rescue both ILC3 defects and disease severity.

**Fig. 5:**
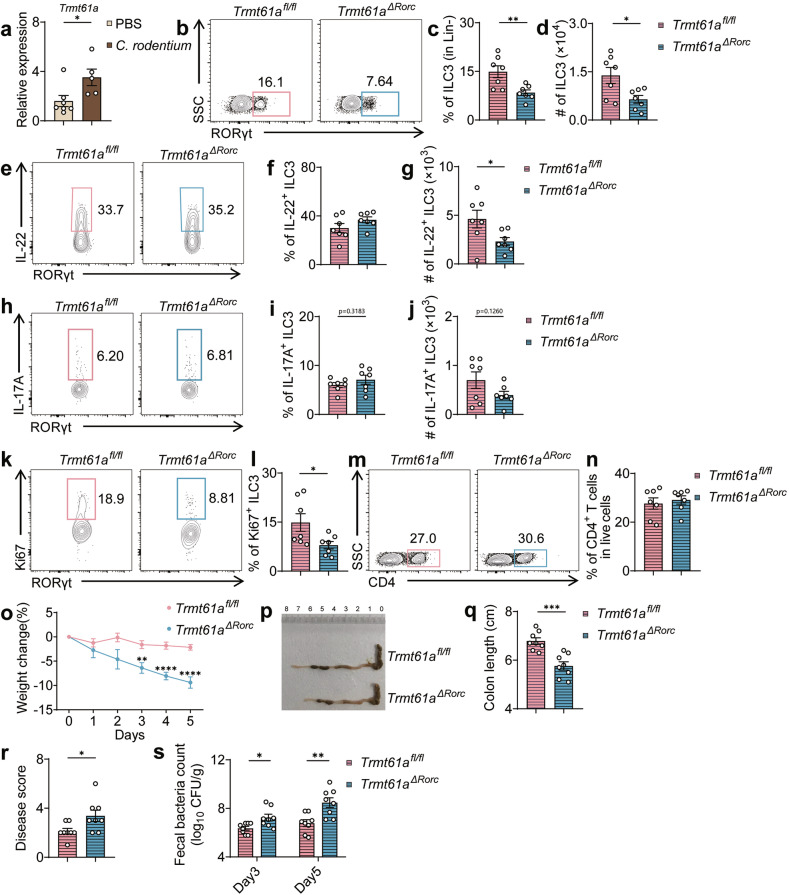
Requirement of TRMT61A for ILC3-Mediated protection against *Citrobacter rodentium* infection. **a** Wild-type mice were orally inoculated with 1 × 10^9^ *Citrobacter rodentium* or PBS as a control. Three days post-infection, intestinal LPLs were isolated. ILC3s (Lin^–^CD45.2^int^CD90.2^hi^) were FACS sorted, and mRNA was extracted. Real-time RT-PCR was conducted to analyze the expression of *Trmt61a* in ILC3s from infected and control mice. *n* = 5–7 mice per group. **b**–**n** *Trmt61a*^*fl/fl*^ and *Trmt61a*^*ΔRorc*^ mice were infected with 1 × 10^9^ *Citrobacter rodentium*. Flow cytometry analysis 5 days post-infection: **b**, **e**, **h**, **k**, **m** Representative flow cytometry plots showing various immune cell populations. Population frequencies of total ILC3s (**c**), IL-22^+^ ILC3s (**f**) and IL-17^+^ ILC3s (**i**). Counts of total ILC3s (**d**), IL-22^+^ ILC3s (**g**) and IL-17^+^ ILC3s (**j**). l Population frequency of Ki67^+^ ILC3s, indicating proliferation. **n** Population frequency of total CD4^+^ T cells. Data from day 5 post-infection, *n* = 7 mice per group. **o**–**r** Morphological and Histological Assessments: changes in body weight (**o**), representative image of the large intestine (**p**), colon length measurements (**q**), histology scores assessing tissue damage (**r**). Data from day 5 post-infection, *n* = 8 mice per group. **s** Bacterial Load Analysis: quantification of bacterial load in feces on days 3 and 5 post-infection. Data are pooled from two independent experiments, presented as means ± SEM. *p < 0.05, **p < 0.01, ***p < 0.001, ****p < 0.0001.

Further, to assess the function of ILC3-specific TRMT61A during bacterial infection, we infected mice with *Citrobacter rodentium*. Post-infection, TRMT61A expression in ILC3s significantly increased, suggesting an active role in defending against *C. rodentium* (Fig. 5a). Consistently, *Trmt61a*^*ΔRorc*^ mice displayed markedly reduced percentages and numbers of total ILC3s and IL-22-producing ILC3s in the colon five days post-infection, compared to controls (Fig. 5b–g), along with a decreasing trend in the numbers of IL-17A-producing ILC3s (Fig. 5h–j). These mice also showed lower percentages of Ki-67^+^ ILC3s, indicating compromised cellular proliferation during infection^44^ (Fig. 5k, l). The proportions of CD4^+^ T cells were unaffected during the infection (Fig. 5m, n). With diminished IL-22-producing ILC3 populations, *Trmt61a*^*Δ Rorc*^ mice experienced more severe weight loss, colonic shortening, and heightened disease pathology, as well as increased fecal bacterial loads compared to control mice (Fig. 5o–s). These findings collectively affirm that TRMT61A is crucial for effective ILC3-mediated host defense against bacterial challenges.

## Discussion

**Fig. 6:**
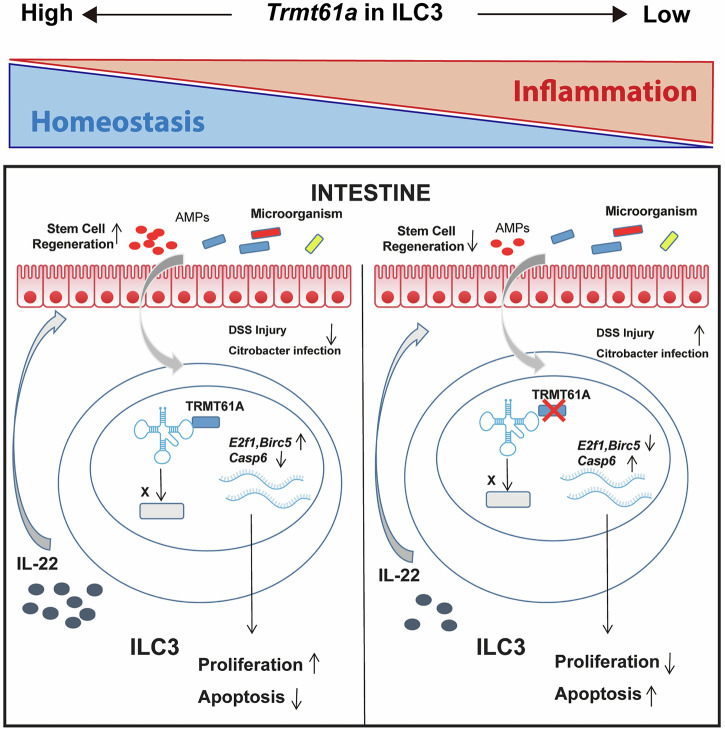
Model illustrating the role of tRNA m^1^A modification in ILC3 homeostasis and function. Proposed model illustrating how tRNA-m^1^A58 modification regulates homeostasis and function of ILC3 in the gut.

Mucosal barriers are continuously challenged by various stimuli, and the maintenance of mucosal barrier homeostasis relies crucially on tissue-resident immune cells such as ILC3s^45,46^. Found predominantly in lymphoid tissues and enriched in the intestine, ILC3s are pivotal in maintaining the commensal microbial community and defending against enteric bacterial infections^30,43,47–49^. Despite extensive research into how ILC3s regulate infections and facilitate tissue repair^30,43,49^, our understanding of their regulation at the epigenetic level, especially concerning RNA modifications, remains incomplete. In this study, we present a model where TRMT61A enhances the proliferation and survival of intestinal ILC3s, thereby protecting mice against experimental intestinal damage and enteric bacterial infections (Fig. 6).

Utilizing RNA-seq and qPCR analyses, complemented by GSEA and GO analyses, we identified significant deficits in cell proliferation and the cell cycle in *Trmt61a*-deficient ILC3s. Specifically, E2F1, a crucial regulator of the cell cycle transition from G1 to S phase^27^, was markedly downregulated, potentially leading to diminished ILC3 proliferation and numbers. Given that TRMT61A influences m^1^A modifications on tRNA, which are critical for protein translation initiation and elongation, the transcriptional downregulation of E2F1 suggests an indirect effect of TRMT61A activity. This highlights the necessity for further studies, such as protein mass spectrometry, to pinpoint TRMT61A’s direct targets in ILC3s. The challenges of acquiring sufficient protein from small numbers of ILC3 cells for mass spectrometry underscore the need for more advanced techniques.

Recent studies have elucidated the profound impact of bacterial colonization and its metabolites on various epigenetic modifications, including those affecting DNA methylation and histone configuration^50–54^. An investigation using Chromatin Immunoprecipitation followed by In Vitro Transcription on ILCs from antibiotic- treated mice revealed significant shifts in H3K4me2 regions^55^, indicating epigenetic alterations induced by microbiota depletion. Our findings of increased *Trmt61a* expression in ILC3s following *C. rodentium* colonization suggest potential alterations in tRNA m^1^A58 modification, which could facilitate rapid protein synthesis crucial for ILC3 activation and proliferation during infection.

Moreover, while microbiota is not essential for the initial development of ILC3s, their absence or alteration — as observed in germ-free and antibiotic-treated mice — significantly reduces NKp46^+^ ILC3 and IL-22-producing ILC3 numbers^56^. The microbial metabolites, particularly SCFAs, are known to enhance ILC3 proliferation through receptors like FFAR2^36^. Together with the Annexin-V/7-AAD staining data, our results suggest that TRMT61A plays a intrinsic role role in maintaining ILC3 numbers. The absence of TRMT61A in ILC3s leads to reduced IL-22^+^ ILC3 numbers, resulting in microbial dysbiosis in *Trmt61a*^*ΔRorc*^ mice. This dysbiosis alters the intestinal microenvironment and further exacerbates the reduction of ILC3s.

This study establishes that TRMT61A expression in ILC3s is crucial for defending against intestinal infection and inflammation. The decrease in the number of IL-22-producing ILC3s in *Trmt61a*^*ΔRorc*^ mice aligns with the observations in patients with inflammatory bowel disease (IBD)^57^. The observed decrease in IL-22-producing ILC3s in *Trmt61a*^*ΔRorc*^ mice correlates with reduced TRMT61A expression levels, suggesting that similar mechanisms may operate in IBD, where inflammation could suppress TRMT61A expression, thereby diminishing ILC3 functionality. Future investigations into TRMT61A’s role in IBD could provide new therapeutic avenues.

In summary, our findings significantly advance the understanding of the biological roles of m^1^A modifications in ILC3s and underscore TRMT61A as a promising target for therapeutic intervention in IBD and related conditions.

## Materials and methods

### Mice

*Trmt61a*^*fl/fl*^ mice were constructed using the CRISPR-Cas9-based genome-editing system by inserting two loxP sites into the loci flanking the first exon, as previously described. *Rorc*^*cre*^ mice were kindle provided by Dr. Lei Shen, Shanghai Institute of Immunology of Shanghai Jiao Tong University, Shanghai. We crossed *Trmt61a*^*fl/fl*^ mice with *Rorc*^*cre*^ mice to obtain *Trmt61a*^*fl/fl*^ *Rorc*^*cre*^ offspring in which the expression of TRMT61A is specifically absent in ILC3s.

Mice used for in vivo studies were littermate controlled. Both male and female mice were used unless otherwise noted. All mice used in this study are on C57BL/6 background, and were maintained in specific pathogen–free facilities and used according to protocols approved by Animal Care and Use Committees of the Shanghai Jiao Tong University School of Medicine.

### Isolation of intestinal lamina propria lymphocytes (LPLs) and intestinal epithelial cells

Small intestine or colon were dissected, fat tissues and peyer’s patches were removed. Intestines were cut open longitudinally and washed in PBS. Intestines were then cut into 3-cm-long pieces, washed, and shaken in PBS containing 1 mM DTT for 10 min at RT. Intestines were incubated with shaking in PBS containing 30 mM EDTA and 10 mM HEPES at 37 °C for 10 min for two cycles. Supernatant from the first round of EDTA was saved as intestinal epithelial cells. The tissues were then digested in the RPMI1640 medium (Thermo Fisher Scientific) containing DNase I (150 µg/mL, Sigma) and collagenase VIII (200 U/mL, Sigma) at 37 °C in a 5% CO_2_ incubator for 1.5 h. The digested tissues were homogenized by vigorous shaking and passed through 100-µm cell strainer. Mononuclear cells were then harvested from the interphase of an 80 and 40% Percoll gradient after a spin at 2500 rpm for 20 min at RT.

### Flow cytometry

Anti-mouse CD16/32 antibody (Biolegend) was used to block the nonspecific binding to Fc receptors before all surface stainings. Dead cells were stained with live and dead violet viability kit (Invitrogen), and were gated out in analysis. For nuclear stainings, cells were fixed and permeabilized using a Mouse Regulatory T Cell Staining Kit (Thermo Fisher Scientific). For detection of IL-22 production in ILC3s, cells were stimulated by PMA (50 ng/mL, Sigma) and ionomycin (500 ng/mL, Sigma) for 4 h. Brefeldin A (2 µg/mL, Sigma) was added for the last 2 h before cells were harvested for analysis.

Antibodies used in this study were listed as follows:

LIVE/DEAD™ Fixable Violet Dead Cell Stain Kit, # L34964, Invitrogen, 1:1000 Zombie Aqua™ Fixable Viability Kit, # 423102, Biolegend, 1:500

CD90 BV605, # 140317, Biolegend, 1:200

CD127 FITC, # 135007, Biolegend, 1:200

NKp46 APC-ef780, # 47-3351-82, eBioscience, 1:200

CCR6 PE, # 129804, Biolegend, 1:200

GATA3 PE, # 653804, Biolegend, 1:40

RORψt APC, # 17-6981-82, eBioscience, 1:200

CD4 AF700, # 100430, Biolegend, 1:200

CD45.2 APC-CY7, # 109824, Biolegend, 1:200

CD45 PerCP-CY5.5, # 103132, Biolegend, 1:200

CD3ɛ PE-CY7, #100220, Biolegend, 1:200

CD3ɛ PerCP, # 100326, Biolegend, 1:200

CD3 BV785, # 100355, Biolegend, 1:200

TCRb PE-Cy7, # 109222, Biolegend, 1:200

CD5 PE-CY7, # 100622, Biolegend, 1:200

FcɛRI PE-CY7, # 134318, Biolegend, 1:200

F4/80 PE-CY7, # 123114, Biolegend, 1:200

CD11b PE-CY7, # 101216, Biolegend, 1:200

CD11c PE-CY7, # 117318, Biolegend, 1:200

CD11b FITC, # 101206, Biolegend, 1:200

CD11c FITC, # 117306, Biolegend, 1:200

Ki67 PE-CY7, # 652426, Biolegend, 1:200

IL-22 PE, # 51640,4 Biolegend, 1:150

### Bone marrow transplantation

Bone marrow cells were isolated from the femur and tibia of *Trmt61a*^*ΔRorc*^, *Trmt61a*^*fl/fl*^ or CD45.1 wildtype mice. Red blood cells were lysed with Red Blood Cell Lysis Buffer (Solarbio), and the remaining cells were counted. A total of 5 × 10^6^ bone marrow cells in 200 μL PBS were intravenously injected into each lethally irradiated CD45.1 wild-type recipient. Eight weeks post-transplantation, intestinal ILC3s were analyzed by flow cytometry.

### Antibiotics treatments

Pregnant mice were treated with a broad-spectrum mixture of antibiotics (ampicillin (1 g/L), vancomycin (0.5 g/L), metronidazole (1 g/L), neomycin (1 g/L), and gentamicin (1 g/L)) in their drinking water. After birth, the pubs were constantly treated with the antibiotics cocktail as describe above until 2 months.

### IL-22 in vivo treatment

For IL-22 treatment, 25 μg recombinant protein (novoprotein) was injected intraperitoneally in a volume of 200 μL PBS for each *Trmt61a*^*ΔRorc*^ mice.

### Fecal microbiota transfer

Fresh fecal pellets were collected from *Trmt61a*^*ΔRorc*^ or *Trmt61a*^*fl/fl*^ donor mice and suspended in sterile PBS. Recipient wild-type mice were pretreated with a broad-spectrum antibiotic cocktail to deplete endogenous microbiota. 200 μL of fecal suspension was administered to each recipient by oral gavage. Recipients were maintained for 6 weeks post-transplantation before analysis of intestinal ILC3 populations by flow cytometry.

### *Citrobacter rodentium* infection model

*Citrobacter rodentium* (DBS100 strain) was cultured overnight and bacterial concentration was calculated by measuring optical density at a wavelength of 600 nm (OD600) with spectrometer. *Trmt61a*^*ΔRorc*^ and *Trmt61a*^*fl/fl*^ mice or WT mice were orally infected with 1 × 10^9^ CFU of *C. rodentium*. Mice were weighed daily. At day 5 after infection, the colon was collected from the infected mice. Colon length was measured. Colon was fixed with 4% paraformaldehyde for histology or used for the isolation of colonic immune cells as describe above. For assessment of bacterial burden, feces were collected at day 3 and day 5 after infection, weighed and homogenized with PBS. The homogenates were plated on MacConkey agar plate and counted after overnight incubation at 37 °C under aerobic conditions.

### DSS-induced colitis model

*Trmt61a*^*ΔRorc*^ and *Trmt61a*^*fl/fl*^ mice were treated with 3% DSS (MP Biomedicals) in the drinking water for 4 days and followed by regular drinking water for 2 days. Body weight was measured daily and mice were euthanized at day 6. Colon length was measured. Colon was fixed with 4% paraformaldehyde for histology or used for the isolation of colonic immune cells or colonic epithelial cells as describe above.

### Histological analysis

Tissues from proximal colon were dissected and fixed with 4% paraformaldehyde. Tissues were then embedded in paraffin, sectioned at 5 µm, and stained with H&E. Sections were then blindly analyzed using the light microscope (Olympus), and scored according to a previously described scoring system^58^. The four parameters used include (i) the degree of inflammatory infiltration in the LP, range 1–3; (ii) Goblet cell loss as a marker of mucin depletion, range 0–2; (iii) mucosal erosion to frank ulcerations, range 0–2; and (iv) submucosal spread to transmural involvement, range 0–2.The severity of inflammation in sections of the colon was based on the sum of the scores in each parameter (maximum score = 9).

### Microbiota sequencing

Fresh mouse fecal samples were collected and microbial DNA was isolated from stools of indicated mice by using QiAamp DNA Stool Mini Kit (QIAGEN) according to manufacturer’s instructions. DNA concentration was checked with Nanodrop (ThermoFisher Scientific). The V4 regions of the bacteria 16S rRNA gene were amplified by PCR using primers 515F-806R. 16S rRNA sequencing was performed by Oebiotech (Shanghai, China).

### RNA-seq analysis

About 1 × 10^4^ ILC3s (Lin^−^CD45.2^int^Thy1.2^hi^) were sorted from the colon of *Trmt61a*^*ΔRorc*^ and *Trmt61a*^*fl/fl*^ mice separately and were lysed in Trizol (Invitrogen). The total RNA was extracted. Biological duplicates were generated for each group. The total RNA sample was digested by DNaseІ (NEB), and purified by oligo-dT beads (Dynabeads mRNA purification kit, Invitrogen), then poly(A) containing mRNA were fragmented into 130 bp with First-strand buffer. First-strand cDNA was generated by N6 primer, First Strand Master Mix and Super Script II reverse transcription (Invitrogen) (reaction condition: 25 °C for 10 min; 42 °C for 40 min; 70 °C for 15 min). Then add Second Strand Master Mix to synthesize the second-strand cDNA (16 °C for 1 h). Purified the cDNA with Ampure XP Beads (AGENCOURT), then combine with End Repair Mix, incubate at 20 °C for 30 min. Purified and add A-Tailing Mix, incubate at 37 °C for 30 min. Then combine the Adenylate 3′ ends DNA, Adapter and Ligation Mix, incubate the ligate reaction at 20 °C for 20 min. Several rounds of PCR amplification with PCR Primer Cocktail and PCR Master Mix were performed to enrich the cDNA fragments. Then the PCR products were purified with Ampure XP Beads (AGENCOURT). The Qualified libraries will amplify on cBot to generate the cluster on the flowcell (TruSeq PE Cluster Kit V3–cBot–HS, Illumina). The amplified flowcell will be sequenced pair end on the HiSeq 2000 System (TruSeq SBS KIT-HS V3, Illumina), read length 50. Reads were mapped to Mouse Genome Assembly GRCm38.p5 by STAR v2.5. Gene and isoform expression quantification was called by RSEM v1.2 with default parameters on GENCODE mouse M16 gene annotation file. Differential expression analysis was performed by Bioconductor package edgeR v3.18.1. Significantly changed genes were chosen according to two criteria: (1) significance level *p* < 0.05; (2) fold change > 2, and were used for gene ontology enrichment analysis using the website of Gene Ontology Consortium (http://www.geneontology.org). Gene set enrichment analysis was performed using GSEA software (Broad Institute). Normalized heatmap was based on the standard score (Z score) and generated with oebiotech cloud platform (https://cloud.oebiotech.com/task/). The standard score of a raw score x is Z = x−μσ, where μ is the mean of the FPKM value of each sample and σ is the standard deviation of the FPKM value of each sample.

### Quantitative real-time RT-PCR

RNA was isolated with Trizol reagent (Invitrogen). cDNA was synthesized using the GoScript™ Reverse Transcription kit (Promega). Real-time PCR was performed using SYBR Green (Bio-rad). Reactions were run with the Bio-Rad CFX384. The results were displayed as relative expression values normalized to β-actin.

**Table:** 

Target	Primer Sequence (5′-3′)
*Trmt61a* Forward	ATGAGTTTCGTGGCATACGAG
*Trmt61a* Reverse	CCTCTGCTGCAAATCACCTT
*Reg3g* Forward	CAAGGTGAAGTTGCCAAGAA
*Reg3g* Reverse	CCTCTGTTGGGTTCATAGCC
*Reg3b* Forward	CCACTCTGGGTGCAGAAC
*Reg3b* Reverse	AATTCGGGATGTTTGCTGTC
*Reg3a* Forward	TCACCTGGTCCTCAACAGTATT
*Reg3a* Reverse	GGAGCGATAAGCCTTGTAACC
*E2f1* Forward	CTCGACTCCTCGCAGATCG
*E2f1* Reverse	GATCCAGCCTCCGTTTCACC
*E2f1* Forward	CTGAGCGAAGTATTGGAGACAG
*E2f1* Reverse	CTGCGGATTGTTTGGCTTCC
*Birc5* Forward	GAGGCTGGCTTCATCCACTG
*Birc5* Reverse	CTTTTTGCTTGTTGTTGGTCTCC
*Casp6* Forward	GGAAGTGTTCGATCCAGCCG
*Casp6* Reverse	GGAGGGTCAGGTGCCAAAAG

### Bacterial DNA extraction and real-time PCR

Fecal pellets were collected and total bacterial DNA was extracted using the Stool DNA Kit (Omega Biotek). Quantitative PCR for detection of specific *Muribaculaceae* strains was performed with SYBR Green (Bio-Rad) and normalized to total bacterial DNA. Reactions were run with the Bio-Rad CFX384. Primers used in this study were shown below^59^.

**Table:** 

Target	Primer Sequence (5′-3′)
*All bacteria*	Forward CGGTGAATACGTTCCCGG
*All bacteria*	Reverse TACGGCTACCTTGTTACGACTT
*Muribaculum intestinale*	3SKV Forward AACAACAACCAGCTGACAAT
*Muribaculum intestinale*	3SKV Reverse AGTAGTTCCACTGGCAGG
*Muribaculum intestinale*	NM65 Forward GTAACAACAACCAGTTGACAT TG
*Muribaculum intestinale*	NM65 Reverse TAATAGTTCCACTGGCATGCA
*Muribaculum intestinale*	NM06 Forward CAACAACAACCAGCTGACTAT G
*Muribaculum intestinale*	NM06 Reverse AATAGTTCCACTGACATGCC
*Muribaculum intestinale*	7GAM Forward AACAACAACCAGTTGACCAT G
*Muribaculum intestinale*	7GAM Reverse TAGTAGTTCCACTGGCAGG
*Muribaculum intestinale*	8CFAA Forward AACAACAACCAGCTGACAAT
*Muribaculum intestinale*	8CFAA Reverse CAACAACAACCAGCTGACA AT
*Muribaculum intestinale*	8FP Forward CAACAACAACCAGCTGACAAT
*Muribaculum intestinale*	8FP Reverse AGTAGTTCCACTGGCAAGC
*Muribaculum intestinale*	6FAAM Forward CAACAACAACCAGCTGACA AT
*Muribaculum intestinale*	6FAAM Reverse TAGTTCCACTGGCATGCC
*Muribaculum intestinale*	16COLB Forward CAACAACAACCAGCTGACA AT
*Muribaculum intestinale*	16COLB Reverse CAATAGTTCCACTGGCATGC

### Statistical analysis

Unpaired Student’s *t*-tests and two-way analysis of variance (ANOVA) were used to compare pairs of groups, and all data are presented as the means ± SEM. *p* values of < 0.05 were considered statistically significant.

## Supplementary information

Supplementary information, Figs. S1–S6

## Data Availability

All data needed to evaluate the conclusions in this article are present in the paper or the supplementary materials (or both). The bulk RNA-seq data generated in this study are available at the Gene Expression Omnibus (GSE267755). The Trm61a flox mouse strain can be provided by H.B.L. pending scientific review and a completed materials transfer agreement. Requests for those mouse lines should be submitted to H.B.L.
