# Supplementary material for: tRNA m1A modification is essential for gut homeostasis and function of group 3 innate lymphoid cells
Source: Cell Discov. 2026 Jan 3;12:1. doi: 10.1038/s41421-025-00850-9 (PMC12764812; doi:10.1038/s41421-025-00850-9)
Supplement: Supplementary file 1 — Supplementary information, Figs. S1–S6 [file 41421_2025_850_MOESM1_ESM.pdf]

Supplementary Figures and Figure lengends

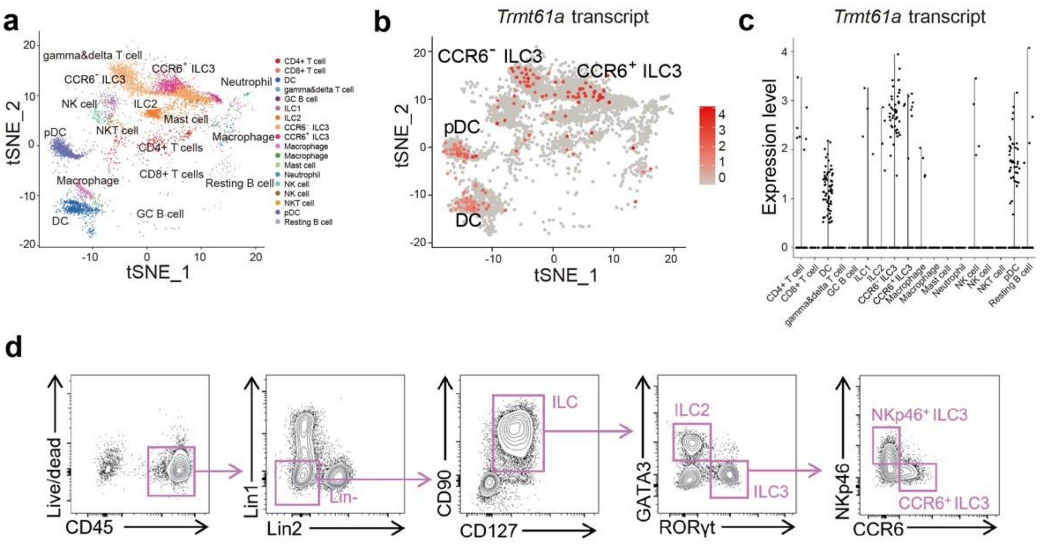

Figure S1. Expression Profile of *Trmt61a* in Intestinal ILC3s

Single-cell RNA sequencing (scRNA-seq) data from mouse small intestinal LPLs were analyzed to examine the expression of *Trmt61a*. Data were retrieved from the public dataset GSE124880 and reanalyzed for this study.

(a) t-SNE plot displaying distinct clusters of intestinal LPLs, illustrating the cellular heterogeneity within the sample.

(b) Expression of *Trmt61a* across different immune cell clusters, visualized on the t-SNE plot to show the specific populations expressing *Trmt61a* at higher levels.

(c) Violin plot detailing the expression levels of *Trmt61a* within each immune cell cluster, highlighting variability and expression intensity.

(d) Gating strategy of ILC3s. Lin, lineage markers. Lineage 1: CD3ε, CD5, FcεRI, F4/80. Lineage 2: CD11b, CD11c and B220. ILC3s were gated as $CD45^{+}Lin^{-}$ $CD127^{+}CD90^{+}ROR\gamma t^{+}$, and further gated as $NKp46^{+}$, $CCR6^{+}$, or DN subset.

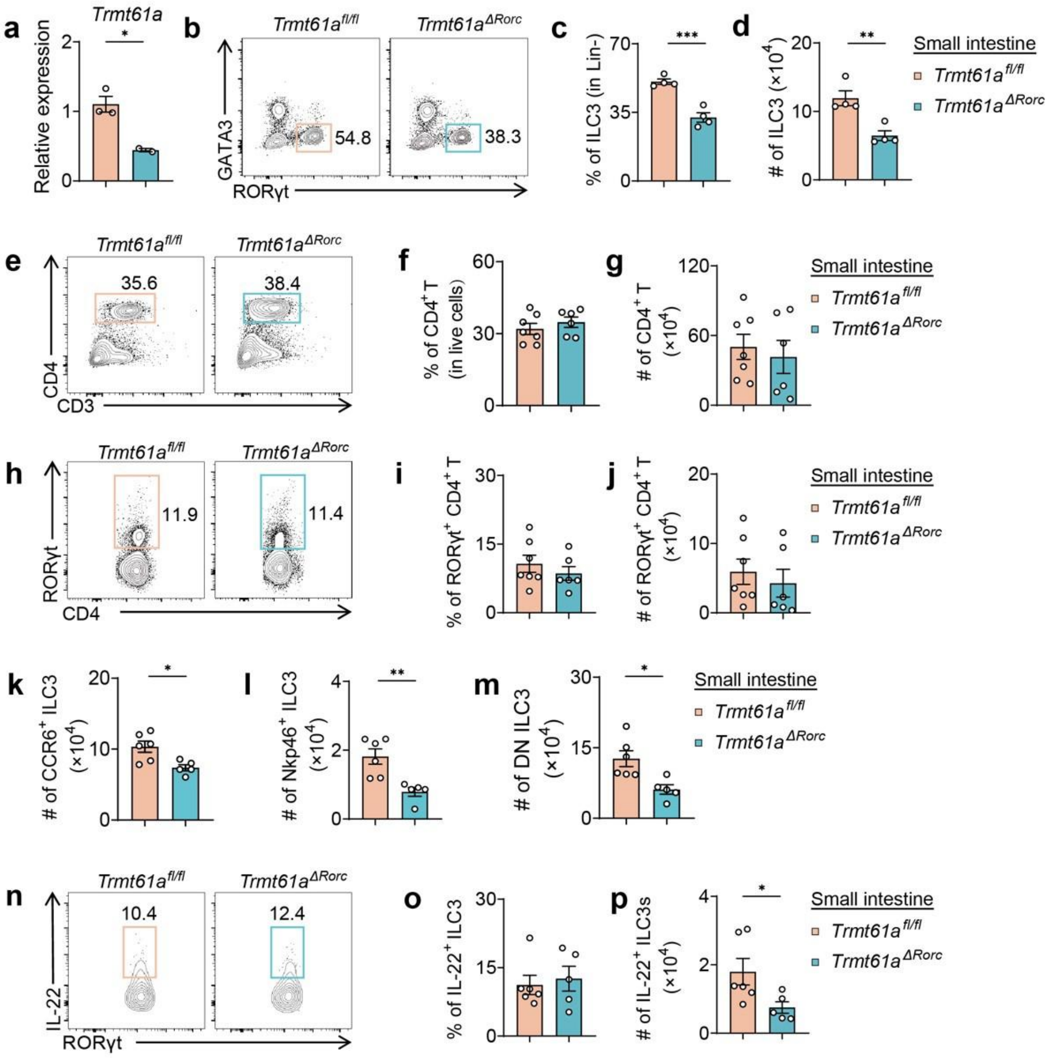

Figure S2. TRMT61A Regulation of ILC3 Homeostasis in the Small Intestine

(a) Real-time RT-PCR was used to measure the mRNA levels of *Trmt61a* in ILC3s from small intestinal LPLs of $Trmt61a^{fl/fl}$ (n=3) and $Trmt61a^{\Delta Rorc}$ mice (n=2).

(b to d) Flow cytometry analysis of ILC3s in small intestinal LPLs: (b) Representative flow cytometry plots. (c) Population frequency. (d) Cell counts. n = 4 mice per group.

(e to j) Flow cytometry analysis of $CD4^+$ T Cells in small intestinal LPLs: (e and h) Representative plots. (f and i) Population frequencies of $CD4^+$ T cells (f) and $ROR\gamma t^+$ $CD4^+$ T cells (i). (g and j) Cell counts. n = 6-7 mice per group.

(k to m) Counts of specific ILC3 subsets in small Intestinal LPLs: (k) $CCR6^+$ ILC3s. (l) $Nkp46^+$ ILC3s. (m) DN (double-negative) ILC3s. n = 5-6 mice per group.

(n to p) IL-22^+ ILC3s analysis in small intestinal LPLs: (n) Representative flow

cytometry plots. (o) Population frequency. (p) Cell counts. n = 5-6 mice per group. Data are pooled from two independent experiments, presented as means ± SEM. *P < 0.05, **P < 0.01, ***P < 0.001.

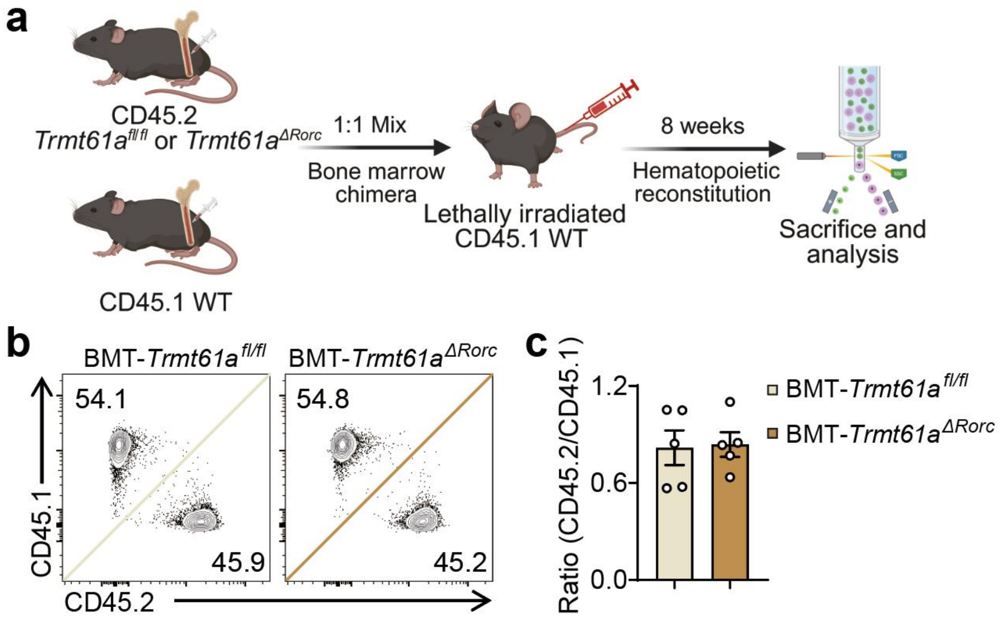

Figure S3. TRMT61A-deficient ILC3s develop normally under competitive conditions.

(a) Schematic of experimental design. CD45.1^{+} WT recipients were lethally irradiated and reconstituted with a 1:1 mixture of CD45.2^{+} *Trmt61a*$^{\Delta Rorc}$ (or *Trmt61a*$^{fl/fl}$) bone marrow cells and CD45.1^{+} WT competitor cells, termed BMT-*Trmt61a*$^{\Delta Rorc}$ and BMT-*Trmt61a*$^{fl/fl,}$ respectively. After 8 weeks, recipient mice were sacrificed for analysis of intestinal ILC3s. n=5 mice per group.

(b) Representative flow cytometry plots showing CD45.2 versus CD45.1 expression in intestinal ILC3s from BMT-*Trmt61a*$^{fl/fl}$ and BMT-*Trmt61a*$^{\Delta Rorc}$ chimeras.

(c) Quantification of CD45.2/CD45.1 ratios in intestinal ILC3s. Data are pooled from two independent experiments, presented as means ± SEM.

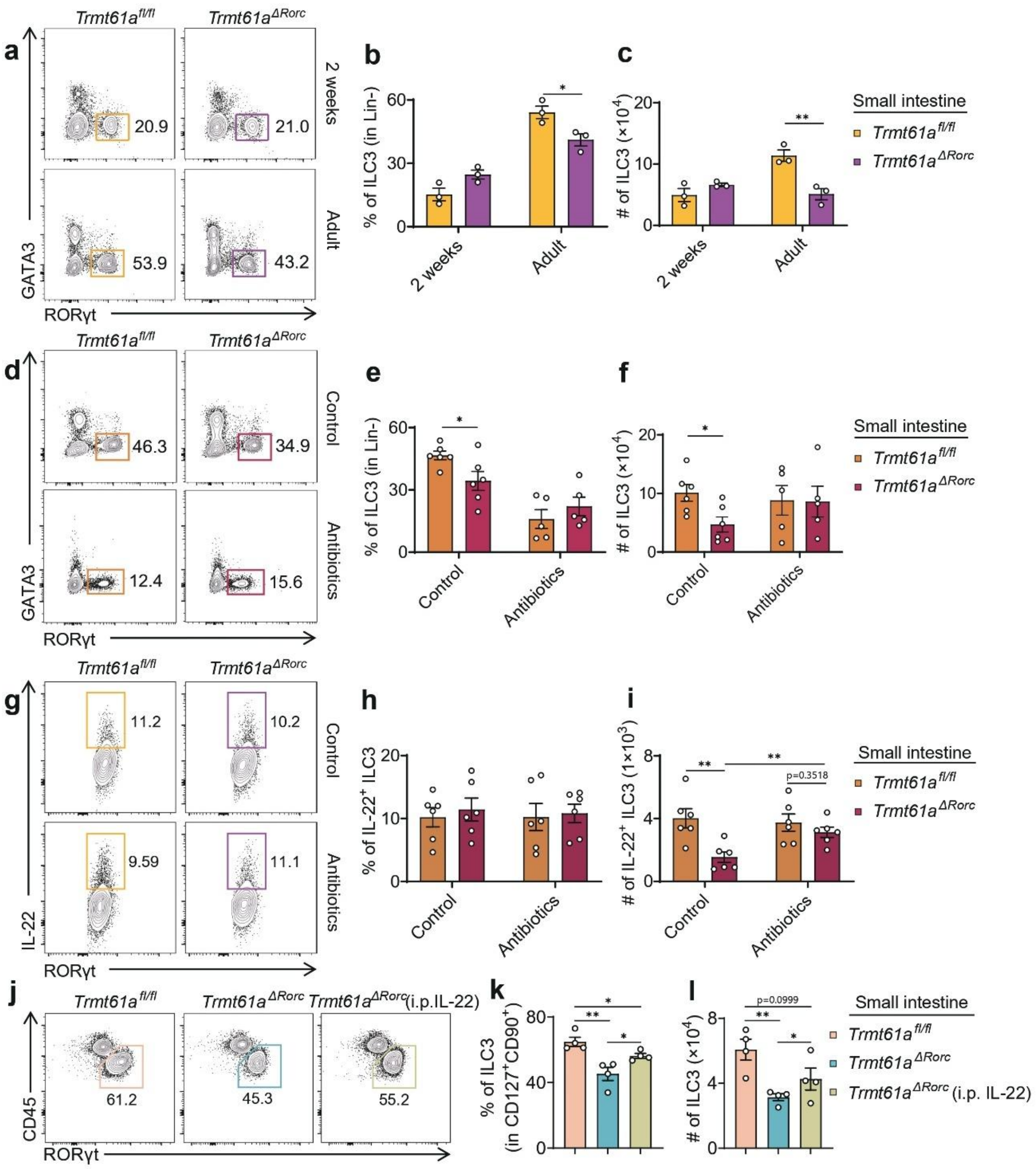

Figure S4. Influence of Microbiota on TRMT61A Regulation in ILC3s in the Small Intestine

(a to c) Flow cytometry analysis of ILC3s from small intestinal LPLs of 2-week-old and adult *Trmt61a*$^{fl/fl}$ and *Trmt61a*$^{\Delta Rorc}$ mice: (a) Representative flow cytometry plots. (b) Population frequency of ILC3s. (c) Cell counts of ILC3s. n = 3 mice per group.

(d to f) Analysis of ILC3s in small intestinal LPLs post-antibiotic treatment administered two months after birth to *Trmt61a*$^{fl/fl}$ and *Trmt61a*$^{\Delta Rorc}$ mice: (d) Representative flow cytometry plots. (e) Population frequency of ILC3s. (f) Cell counts

of ILC3s. n = 5 mice per group.

(g to i) Analysis of IL-22^{+} ILC3s in the small intestine of *Trmt61a*$^{fl/fl}$ and *Trmt61a*$^{\Delta Rorc}$ mice with or without antibiotic treatment: (g) Representative flow cytometry plots. (h) Population frequency of IL-22^{+} ILC3s. (i) Cell counts of IL-22^{+} ILC3s. n = 6 mice per group.

(j to l) Analysis of ILC3 in the small intestine of *Trmt61a*$^{fl/fl}$ mice, *Trmt61a*$^{\Delta Rorc}$ mice, and *Trmt61a*$^{\Delta Rorc}$ mice with intraperitoneal IL-22 administration: (j) Representative flow cytometry plots. (k) Population frequencies of ILC3s. (l) Counts of ILC3s. n =4 mice per group.

Data are presented as means ± SEM. *P < 0.05, **P < 0.01.

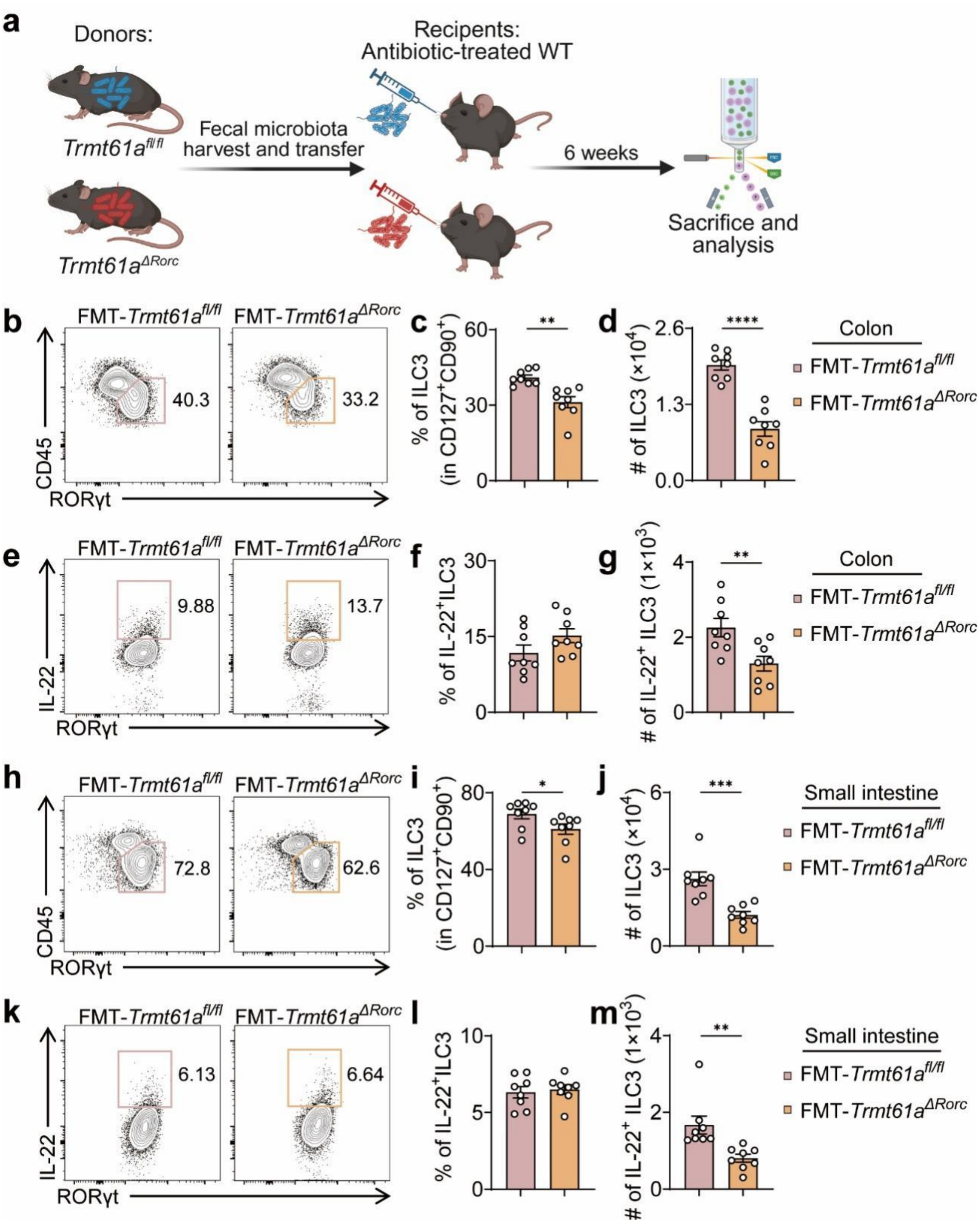

Figure S5. Dysbiotic microbiota from *Trmt61a*$^{\Delta Rorc}$ mice impairs intestinal ILC3s in wild-type recipients

(a) Schematic of fecal microbiota transfer (FMT) from *Trmt61a*$^{fl/fl}$ and *Trmt61a*$^{\Delta Rorc}$ donors into antibiotic-treated wild-type mice, followed by analysis 6 weeks post-transplantation. n = 8 mice per group.

(b to g) Flow cytometry analysis of ILC3s and IL-22^{+} ILC3s in colonic LPLs: (b and e) Representative plots. (c and f) Population frequency. (d and g) Cell counts.

(h to m) Flow cytometry analysis of ILC3s and IL-22$^+$ ILC3s in small intestinal LPLs: (h and k) Representative plots. (i and l) Population frequency. (j and m) Cell counts. Data are pooled from two independent experiments, presented as means ± SEM, *P < 0.05, **P < 0.01, ***P < 0.001, and ****P < 0.0001.

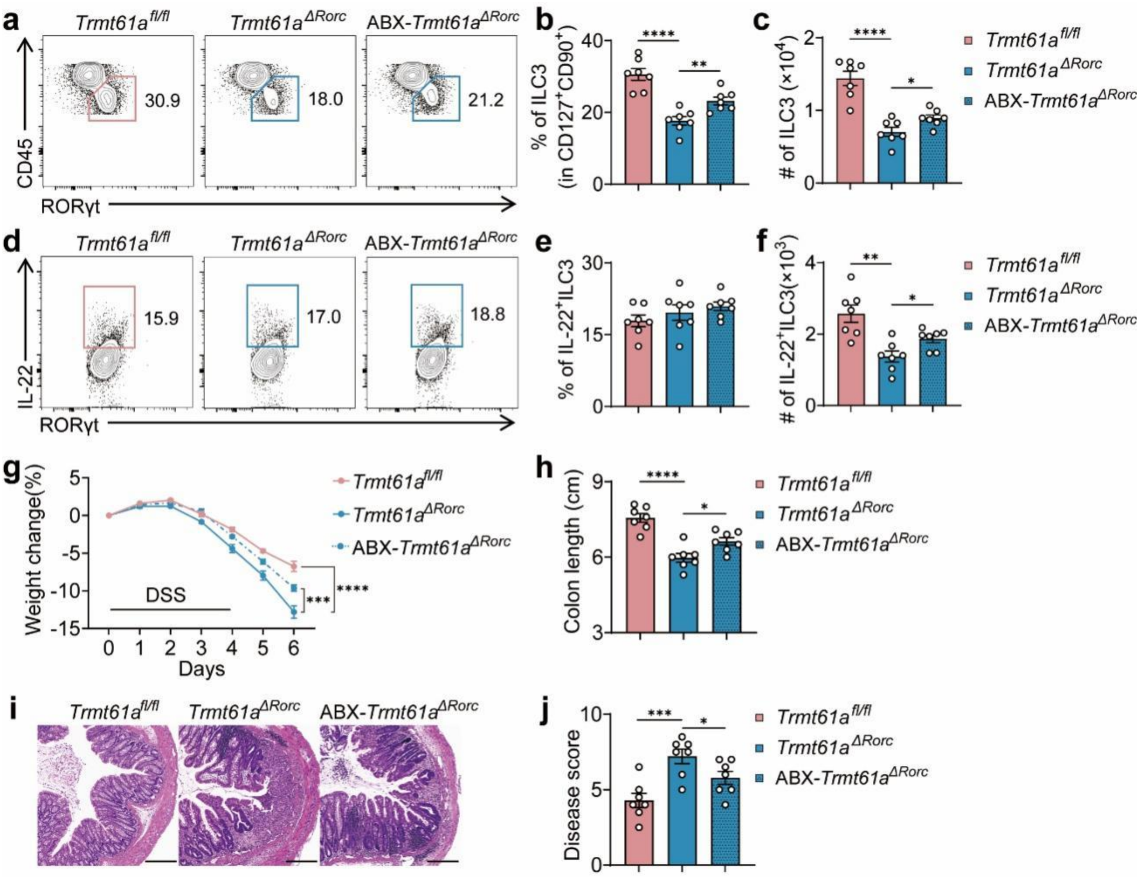

Figure S6. Antibiotic treatment partially restores ILC3s and attenuates DSS-induced colitis in *Trmt61a$^{\Delta Rorc}$* mice

(a to f) Flow cytometry analysis of ILC3s and IL-22^{+} ILC3s in the colon of *Trmt61a$^{fl/fl}$* mice, *Trmt61a$^{\Delta Rorc}$* mice, and antibiotic-treated *Trmt61a$^{\Delta Rorc}$* mice (termed ABX- *Trmt61a$^{\Delta Rorc}$*): (a and d) Representative flow cytometry plots showing total ILC3s (a) and IL22^{+} ILC3s (d). (b and e) Population frequencies of total ILC3s (b) and IL-22^{+} ILC3s (e). (c and f) Counts of total ILC3s (c) and IL-22^{+} ILC3s (f).

(g to j) Morphological and histological assessments: (g) Body weight changes. (h) Measurements of colon length. (i) Representative images of colon sections. Scale bars represent 250 μm. (j) Disease score. n = 7 mice per group. Data are pooled from two independent experiments, presented as means ± SEM, *P < 0.05, **P < 0.01 and ***P < 0.001.
